# Stakeholder engagement from problem analysis to implementation strategies for a patient‐reported experience measure in disability care: A qualitative study on the process and experiences

**DOI:** 10.1111/hex.13147

**Published:** 2020-10-30

**Authors:** Marjolein van Rooijen, Stephanie Lenzen, Ruth Dalemans, Anna Beurskens, Albine Moser

**Affiliations:** ^1^ Department of Family Medicine CAPHRI School for Public Health and Primary Care Maastricht University Maastricht the Netherlands; ^2^ Research Centre for Autonomy and Participation of Persons with a Chronic Illness Zuyd University of Applied Sciences Heerlen the Netherlands

**Keywords:** disability care, implementation science, participatory research, stakeholder participation

## Abstract

**Background:**

In implementation science, vast gaps exist between theoretical and practical knowledge. These gaps prevail in the process of getting from problem analysis to selecting implementation strategies while engaging stakeholders including care users.

**Objective:**

To describe a process of how to get from problem analysis to strategy selection, how to engage stakeholders, and to provide insights into stakeholders’ experiences.

**Design:**

A qualitative descriptive design.

**Setting and participants:**

The setting was a care organization providing long‐term care to people with acquired brain injuries who are communication vulnerable. Fourteen stakeholders (care users, professionals and researchers) participated. Data were collected by a document review, five interviews and one focus group. Inductive content analysis and deductive framework analysis were applied.

**Intervention:**

Stakeholder engagement.

**Main outcome measures:**

A three‐step process model and stakeholders experiences.

**Results and conclusion:**

We formulated a three‐step process: (a) reaching consensus and prioritizing barriers; (b) categorizing the prioritized barriers and idealization; and (c) composing strategies. Two subthemes continuously played a role in how stakeholders were engaged during the process: communication supportive strategies and continuous contact. The experiences of stakeholder participation resulted in the following themes: stakeholders and their roles, use of co‐creation methods and communication supportive strategies, building relationships, stimulus of stakeholders to engage, sharing power, empowerment of stakeholders, feeling a shared responsibility and learning from one another. We conclude that the inclusion of communication‐vulnerable care users is possible if meetings are prepared, communication‐friendly presentations and reports are used, and relationship building is prioritized.

## 1 INTRODUCTION

Health‐care research results are often sparsely translated into practice. Implementation science is increasing in popularity, yet there is often still a considerable gap between research and practice.^1^ Implementation models start with a problem analysis to identify current implementation processes, barriers and facilitators for the intervention uptake.^2^, ^3^ Then, identified barriers are prioritized, and suitable implementation strategies are selected.^2^ Within the whole process, those who have a stake in the intended intervention should be involved. Stakeholder engagement is defined as 'interactions between researchers and knowledge users that may vary in intensity, complexity, and level of engagement depending on the nature of the research, the findings, as well as the needs of the particular knowledge user'.^4^ Stakeholder engagement is indispensable and highly encouraged in implementation processes.^5^, ^6^, ^7^ It is thought to improve the quality of the implementation because it adds knowledge on clinical applications, behaviours of care users and professionals, and organizations’ mechanisms.^8^, ^9^, ^10^

The study presented here is part of a research project aiming to improve the implementation of a narrative Patient‐Reported Experience Measure (PREM) in disability care while engaging relevant stakeholders. PREMs are defined as 'a measure of patients’ (also read: clients and care users) perceptions of their personal experiences of the healthcare they have received'.^11^ The PREM used in this research project is *‘Dit vind ik ervan!’* (English*: ‘How I feel about it’*), which uses a dialogue between a care professional and a care user to measure the care users’ experiences with the quality of care.^12^ In the first project phase, the current state of the art of the implementation of this PREM was explored, using the Consolidated Framework for Implementation Research.^13^, ^14^ Identified barriers were the following: a top‐down decision to implement the PREM, challenges performing the PREM tailored to (communication vulnerable) care user’s needs, lack of preparation by both care users and care professionals, and care users not feeling safe to share their stories.^14^

Therefore, in this study we focus on prioritizing these barriers and selecting strategies, which aim to improve the PREM implementation while engaging all relevant stakeholders. Relevant stakeholders are care users, care professionals, managers and quality advisors. Engaging care users with acquired brain injury (ABI) as stakeholders is extremely challenging: they are communication vulnerable as a long‐term impact of their ABI. Disabilities from ABI’s are often aphasia and memory loss, psychological and behavioural problems, and physical problems such as epilepsy and fatigue.^15^

These aforementioned challenges complicate care users’ participation as stakeholders because this requires them to understand and comprehend complex information and react instantly in meetings where multiple stakeholders participate.^15^

Besides this challenge, we encountered a twofold problem while conducting the study. First, the process of getting from problem analysis to selecting implementation strategies is not frequently reported in the scientific literature.^16^ Several studies using stakeholder engagement and implementation science in disability care have been published.^17^, ^18^, ^19^, ^20^, ^21^, ^22^ Little is known about how care users can participate in a stakeholder group in a prioritization and selecting process. Descriptions of practical methods to put this process into practice and representations of communications with vulnerable care users in stakeholder groups are very limited.^17^, ^18^, ^20^, ^23^ Thus, clear guidance on how to translate barriers into strategies is needed.^3^

The process of getting from problem analysis to implementation strategies, and how to engage stakeholders, remains a black box.^3^, ^24^ Second, little is written about stakeholders’ experiences with engagement in implementation processes.^25^ Consequently, little is known about stakeholders’ motivations, their needs and preferences for engagement, and their subjective evaluation of their impact.^9^ Having greater insight into stakeholders’ experiences might provide more guidance for applying stakeholder engagement in implementation processes.

Therefore, this study aims to describe a systematic and detailed process of how to get from problem analysis to selecting strategies, how stakeholders can be engaged in this group process, and offers insights into stakeholders’ experiences. The study is relevant because it might support future implementers in applying the implementation process while engaging stakeholders in a valuable manner.

To reach this aim, we answer the following research questions:

- How to get from problem analysis to selecting implementation strategies for a PREM in disability care by engaging all relevant stakeholders?
- How do stakeholders experience their engagement in the process from problem analysis to selecting implementation strategies?

## 2 METHODS

### 2.1 Design

We used a descriptive qualitative study design. This design has the potential to describe a poorly understood phenomenon from the perspective of participants and to answer questions about process factors, for example who, what and where.^26^

### 2.2 Context

The study occurred at a Dutch disability care organization, "*Stichting Gehandicaptenzorg Limburg'* (*SGL*) (English: *Disability Care Foundation Limburg*). SGL offers supported living and living arrangements to people with severe (acquired) intellectual and developmental disabilities, particularly people with acquired brain injuries (ABI). SGL also provides day‐care services for in‐ and outpatients that use SGL facilities.

### 2.3 Participants

We composed a project group aiming to improve the implementation of ‘*Dit vind ik ervan!’* at SGL. Stakeholders in the project group included care user representatives (n = 3), professionals working at SGL (n = 5) and researchers (n = 6). We specifically sampled stakeholders that (a) were able to communicate (with or without the use of communication supportive tools), (b) were able to be a care user with ABI or have experiences with people with ABI, and (c) be willing to work on the implementation of the PREM in collaboration with others. Exclusion criteria were being illiterate or physically unable to attend meetings. We recruited care user representatives by disseminating a communication‐friendly vacancy profile across the care user advisory board of SGL and the university network. Even though three care representatives participated initially, only one representative was able to engage in the whole process due to health issues. We recruited professionals using SGL contacts: two care professionals/PREM trainers, a team leader, a general quality manager and a quality of care policy advisor. The engaged researchers were all part of the research team: a professor, three senior researchers and a junior researcher. (see Table 1).

**TABLE 1:** Characteristics of stakeholders in the project group

Acronym	Background	Age (years)	Gender
Care user representative (1)	Representative of care user board of SGL, suffering from ABI	43	Female
Care user representative (2)	Representative and secretary of care user board of SGL, suffering from ABI	63	Female
Care user representative (3)	Expert in living with ABI (not related to SGL)	64	Male
Care professional 1	Care professional and ‘*Dit vind ik ervan!*’ trainer at SGL	51	Female
Care professional 2	Care professional and ‘*Dit vind ik ervan!*’ trainer at SGL	51	Female
Team leader	Team leader at three SGL facilities	46	Male
General quality manager	General manager at SGL, with a focus on the quality of care, managing all districts of SGL	54	Female
Quality of care policy advisor	Policy advisor at SGL, with a focus on the quality of care	45	Female
Researcher (1)	Professor of goal‐oriented measurement, background in health sciences and physical therapy	53	Female
Researcher (2)	Senior researcher, background in health sciences and occupational therapy	33	Female
Researcher (3)	Senior researcher, background in health sciences and nursing	48	Female
Researcher (4)	Senior researcher, background in health sciences and speech therapy	45	Female
Researcher (5)	Junior researcher, background in health sciences	29	Female

### 2.4 Data collection and procedure

Data were collected between September 2018 and June 2019 via documents (n = 35), individual semi‐structured interviews (n = 5) and one focus group.

#### 2.4.1 Documents

Between August 2018 and February 2019, six project group meetings took place. We collected 35 documents to identify details on which steps were taken and why these were taken, and to describe how stakeholder engagement was applied. Documents included notes of meetings to prepare project group meetings (n = 16), notes of the project group meetings (n = 6) and notes of reflection meetings after project group meetings (n = 13).

#### 2.4.2 Interviews and focus group

Between April and June 2019, one researcher (MvR) conducted semi‐structured interviews (n = 5) with one care user representative, both care professionals/PREM trainers, the quality manager and the quality of care policy advisor of the project group, exploring stakeholders’ experiences with being engaged in the process. We conducted semi‐structured interviews because of their flexibility to improvise in‐depth questions based on participants’ answers.^27^

For the interviews, the participants must have attended at least three project group meetings. All engaged researchers participated in a focus group, which was moderated by a researcher not involved in this study. One interview guide was developed and used for the interviews and the focus group (see Table 2). The interview guide was based on elements of stakeholder engagement, as suggested in a scoping review by Camden et al^5^ The interviews and focus group discussion lasted between 32 and 58 minutes. Even though patterns were shown, and no new findings of the phenomenon were identified we did not reach data saturation because of the inclusion of only one care user as respondent for the interviews.

**TABLE 2:** Interview guide based on a review by Camden et al.^5^

Nr	Interview guide
1.	Strategies for stakeholder engagement
	‐ Expectations of the process
	‐ Identifying project group composition
	‐ Roles and committees
	‐ Supporting stakeholders
2.	Factors influencing engagement
	‐ Communication/culture
	‐ Power‐sharing/empowerment
	‐ Time funding and resources
3	Impacts related to stakeholder engagement
	‐ Creating partnerships and building value
	‐ Accessibility of knowledge and used methods
	‐ Evaluating impacts

### 2.5 Data analysis

To answer the first research question, we analysed notes of meetings to prepare project group meetings (n = 16), notes of project group meetings (n = 6) and notes of reflection meetings after project group meetings (n = 13). We used inductive content analysis,^28^ because of the limited knowledge available about the process of getting from problem analysis to selecting implementation strategies using stakeholder engagement. Two researchers (MvR, SL) read the documents multiple times, and important passages of the documents were assigned with codes representing the meaning of the data. The codes were then clustered into who‐what‐where and categorized into sub categories, generic categories and main categories, making a process model. (MvR, SL, AM, AB) (see Table 3 in results paragraph). To answer the second research question, we used deductive analysis applying the framework method using a pre‐defined analytical framework based on a review by Camden et al^5^ The framework method was used because it can produce structured output and provides a holistic and descriptive view of the data, thereby building on previous knowledge.^29^ The framework method is an approach in which a matrix is formed by systematically analysing and reducing data by case and code. In the first stage—*transcription*, one researcher transcribed the audiotaped interviews and the focus group verbatim. In the second stage—*familiarization with the interview and focus group*, two researchers (MvR, SL) thoroughly read all transcripts and documents to familiarize themselves with the data. In the third stage—*coding*, these researchers independently applied the deductive pre‐defined codes of an analytical framework to relevant text fragments of 10 data sources (five documents and all transcripts) based on a review by Camden et al^5^ In stage four—*developing a working analytical framework*, an alignment session was held between the two researchers to compare the codes and refine the analytic framework. Next, the framework was discussed among the research team, and an agreement was reached on any refinements. In the fifth stage—*applying the analytical framework*, the analytical framework was applied by indexing subsequent transcripts and documents using the existing codes. The framework was iteratively fine‐tuned until all documents and interviews were coded. In stage six—*charting data into the framework matrix*, the final framework was placed in NVivo (version 12) using the quotes of relevant text fragments (the themes are shown in Table 4). In stage seven—*interpreting the data,* data were interpreted and are presented in the results section.

**TABLE 3:** Overview of the three‐step process model and who, what, where and outcomes

Three‐step process	Who	What	Where	Outcomes
Reaching consensus and prioritizing barriers	First meeting: 2 care user representatives 2 professionals/PREM trainers 1 general quality manager 1 quality of care policy advisor 4 researchers	The wall walk to present outcomes of the problem analysis to discuss their interpretation.	Reaching consensus on identified outcomes barriers to safeguard stakeholders’ support.	28 barriers
	Second meeting: 1 care user representative 2 professionals/PREM trainers 1 general quality manager 1 quality of care policy advisor 3 researchers	The MoSCoW method for importance and feasibility ranking (scale 1‐5).	Prioritize barriers based on importance and feasibility to increasing potential success of strategies.	12 prioritized barriers
Categorizing the prioritized barriers and idealization	Third meeting: 1 care user representative 2 professionals/PREM trainers 1 quality of care policy advisor 3 researchers	Using a mind map to visualize and discuss the relation of prioritized barriers.	Categorize barriers through understanding the root causes of the barriers and identifying relations.	Four directions of solution
	Fourth meeting: 1 care user representative 2 professionals/PREM trainers 1 team leader 1 quality of care policy advisor 2 researchers	Describing ideal situations for directions of solution per stakeholder group.	Idealize directions of solution to uncover stakeholder specific ideals about directions of solution.	Shared implementation targets
Composing strategies	Fifth meeting 1 quality of care policy advisor 2 researchers	Compose strategies by analysing notes of previous meetings, and linking these with theoretical concepts.	To explore strategy possibilities fitting the formulated shared implementation targets.	12 possible strategies
	Sixth meeting 1 care user representative 2 professionals/PREM trainers 1 general quality manager 1 quality of care policy advisor 3 researchers	Presentation of potential strategies using PowerPoint and group discussion.	To select draft strategies in discussion with all stakeholders to safeguard their support for further development.	10 draft implementation strategies to be further developed

**TABLE 4:** Themes and subthemes of stakeholder experiences

Theme	Subtheme
Strategies to engage	Stakeholders and their roles
	Use of co‐creation methods and communication supportive strategies
	Building relationships
Factors influencing engagement	Stimulus of stakeholders
	Sharing power
Impact of engagement	Empowerment of stakeholders
	Feeling of shared responsibility
	Learning from one another

### 2.6 Trustworthiness

Trustworthiness was safeguarded by transferability.^30^ We provided information on the context in which the research took place, the data collection procedures and the analysis. To establish credibility, we used triangulation and member checking. In monthly research‐team meetings, researcher triangulation occurred, as the meetings provided opportunities to reflect on methodological issues, as well as on organizational matters (investigator triangulation). We used several data sources documents to reflect upon the process, and interviews to reflect upon stakeholders’ experiences. Researchers identified themselves as stakeholders too, and their reflection was included using a focus group discussion, using the same topic guide as the one used during the interviews (method triangulation). The member check was done by returning summaries of the interviews and focus groups to participants to check for accuracy and resonance with their experiences.^31^

### 2.7 Ethics

Participants received written and verbal information. For the interviews, participants gave written informed consent. We assured participants that data would be dealt with confidentiality. Anonymity was secured by code numbering the data. The study was reviewed and approved by the ethics committee of Zuyderland‐Zuyd (METCZ2019006).

## 3 RESULTS

In the results section, first the research question 'How to get from problem analysis to selecting implementation strategies for a PREM in disability care by engaging all relevant stakeholders?' will be answered. The inductive analysis resulted in a three‐step process model including ‘how were stakeholders engaged’, ‘who was engaged’‐‘what happened’‐‘where in the steps’ and with ‘what outcomes’. Second, we answer the research question 'How do stakeholders experience their engagement in the process from problem analysis to selecting implementation strategies?'. The framework analysis led to identification of the subthemes ‘stakeholders and their roles’, ‘use of co‐creation methods and communication supportive strategies’, ‘building relationships’, ‘stimulus of stakeholders to engage’, ‘sharing power’, ‘empowerment of stakeholders’, ‘feeling a shared responsibility’ and ‘learning from one another’.

### 3.1 'How to get from problem analysis to selecting implementation strategies for a PREM in disability care by engaging all relevant stakeholders?'

#### 3.1.1 Process model steps and ‘Who was engaged’‐‘what happened’‐‘where in the steps’ and with ‘what outcomes’

Table 3 displays the process model. Table 3 provides an overview of who, what, where and outcomes of meetings.

##### Step 1: Reaching consensus and prioritizing

**FIGURE 1:**
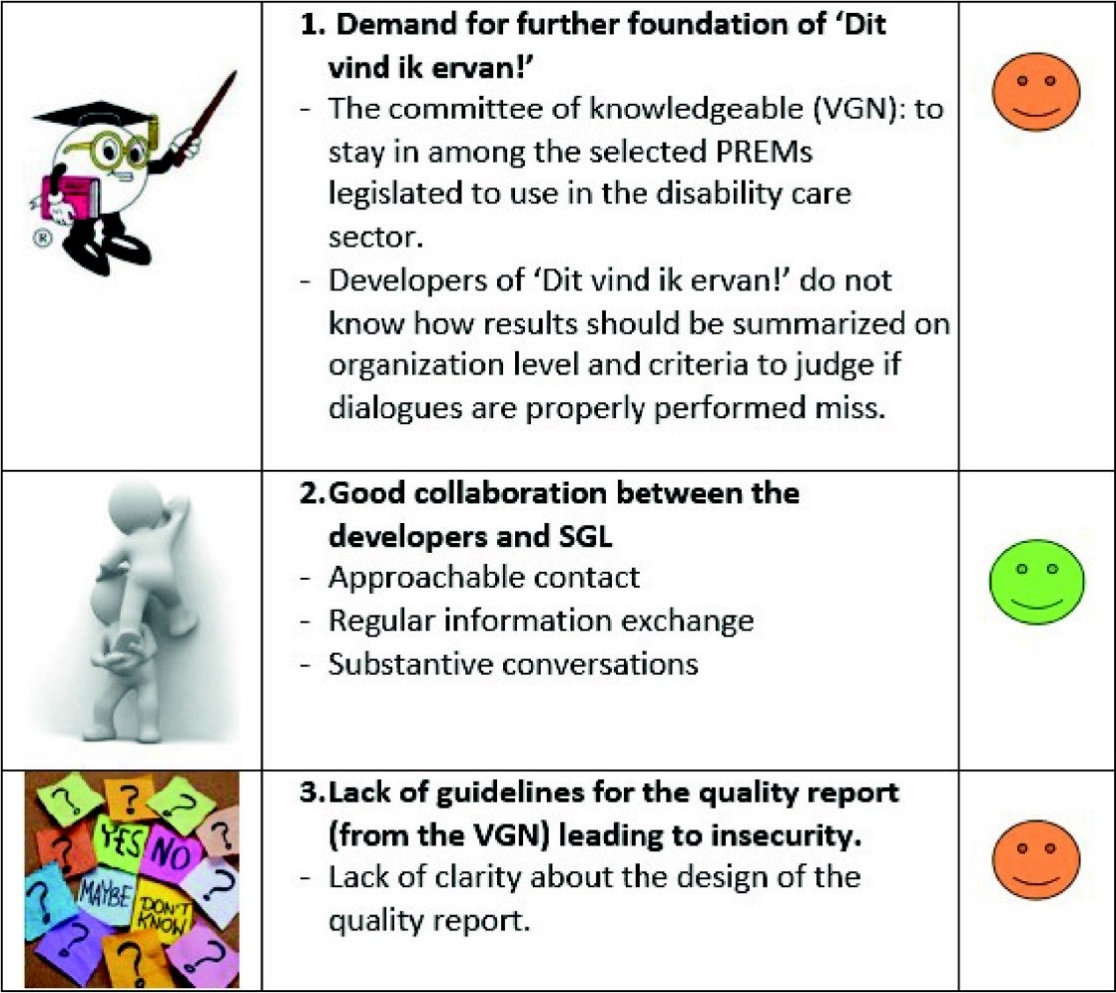
Example of presentation of the 35 findings

In this step, care user representatives, professionals/PREM trainers, a general quality manager, a quality of care policy advisor and researchers participated. In the first meeting, we used the wall walk method^31^ in which several posters visually represented the barriers (see Figure 1). The 35 findings were visualized and categorized using coloured smileys (green for facilitators, orange for factors seen as both facilitators and barriers, and red for barriers), which facilitated the rapid understanding of each result by the stakeholders. The wall walk method enabled stakeholders to read the findings at their reading tempo. We wanted to reach a consensus about the implementation barriers identified in the first phase of the project in order to safeguard stakeholders’ support for the upcoming process. Then, findings were discussed and reframed according to the stakeholders’ viewpoints, resulting in a consensus on 28 barriers. In the second meeting, we used the MoSCoW method, whereby stakeholders had to choose whether barriers must, should, could or would be important to be solved, by putting stickers with written barriers in one of the four options.^32^ Only barriers placed in the 'must' and 'should' option by at least three stakeholders, were scored for feasibility (scale 1‐5, 1 no importance and 5 highest importance). We wanted to increase potential success of strategies focusing on most important and feasible barriers. The outcomes of the second meeting were 12 prioritized barriers.

##### Step 2: Categorizing and idealizing

**FIGURE 2:**
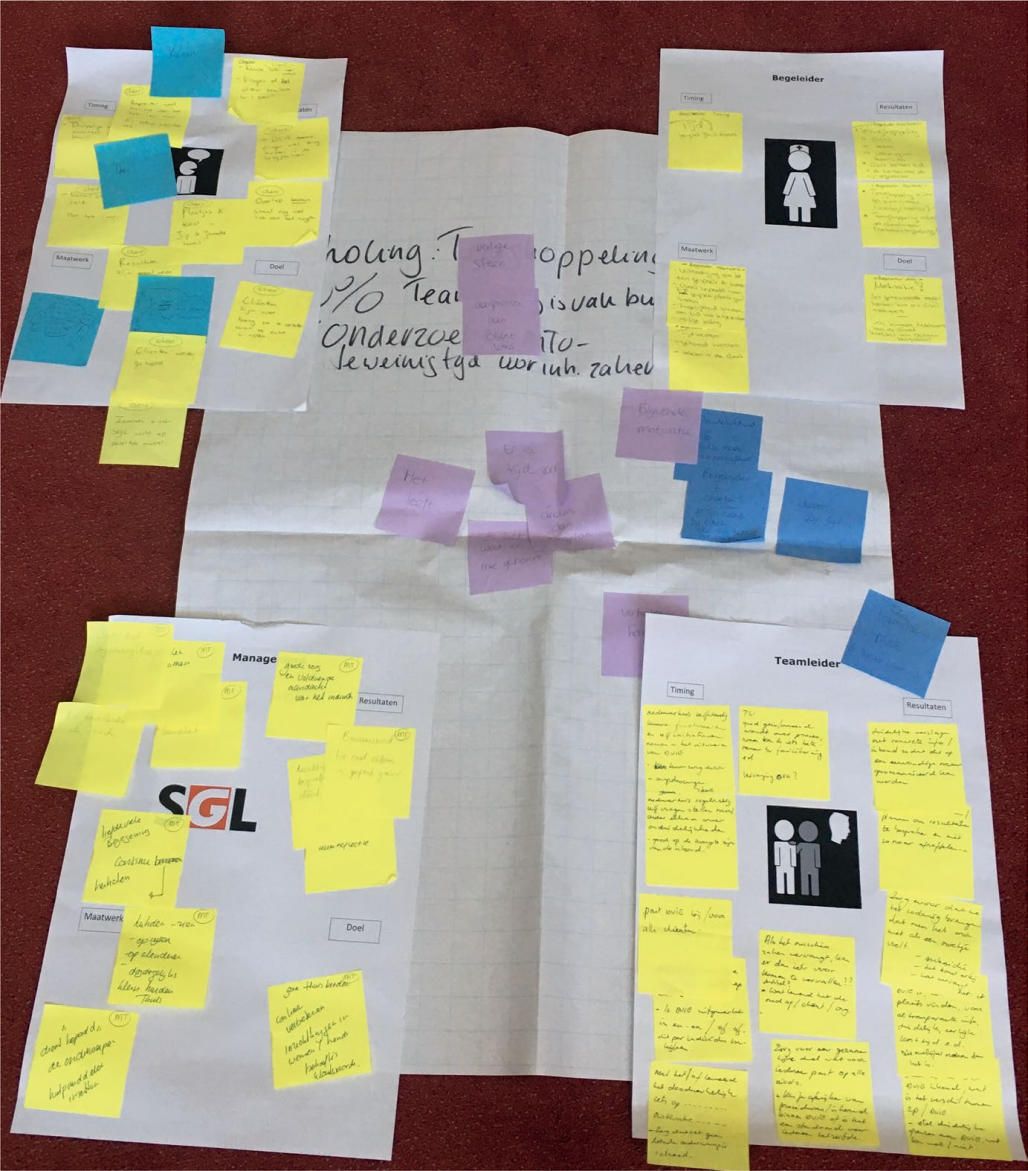
Picture of ideal situations

In this step, a care user representative, professionals/PREM trainers, a quality of care policy advisor and researchers participated. In the third meeting, we used a group discussion led by one researcher using pen and paper to create a mind map, to visualize and discuss the relation of the prioritize barriers. We did this to understand the root causes of the prioritized barriers and to categorize them into four global directions of solution that could potentially tackle multiple barriers. In the fourth meeting, for all four directions of solution stakeholder groups described their ideal situation on A3 size paper. At the end of the meeting, the ideals for directions of solution were shared and discussed. We did this to create a shared vision of the intended outcomes of the implementation process. Figure 2 shows an picture with an example of an ideal situation.

##### Step 3: Composing strategies

In this step, a care user representative, professionals/PREM trainers, a general quality manager, a quality of care policy advisor and researchers participated. They composed possible strategies by analysing notes of previous meetings, and linking these with practical experience and theoretical concepts. We did this to explore strategy possibilities and generate ideas for implementation strategies fitting the formulated implementation targets. The meetings outcomes were a selection of 12 possible implementation strategies. With a PowerPoint presentation, the 12 possible strategies were explained, followed by a discussion on the suitability of the strategies among the stakeholders. This was done to safeguard all stakeholders’ support for the implementation strategies to be further developed. The outcomes were 10 promising implementation strategies.

Two subthemes were identified to continuously play a role in how stakeholders were engaged during the process: communication supportive strategies and continuous contact.

##### Communication supportive strategies

**FIGURE 3:**
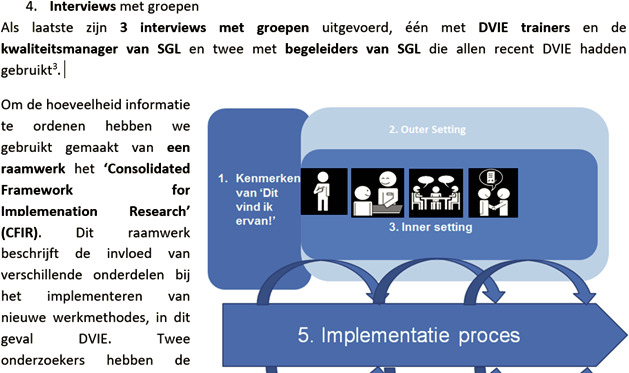
Example of a meeting report

To facilitate the valuable engagement of all stakeholders, communication supportive strategies were used in all the meetings. For written information (eg meeting reports of presentations used during the meetings), we used language‐based strategies, such as the use of short sentences (max 10 words per sentence), high frequently used words and one message per sentence. Moreover, we used visual strategies, such as bright colours, drawings, photographs, pictos and smileys. (see Figure 3).

##### Continuous contact

Before the start of the project, one researcher met individually with all stakeholders for a face‐to‐face introduction to the project. To provide extra support, this researcher met with the care user representatives before every project group meeting to prepare the meeting together. The researcher explained the content of the meeting using oral and visual information and provided room for questions. Moreover, in case a stakeholder could not attend a meeting, the researcher contacted the stakeholder to explain what was done during the meeting to safeguard that all stakeholders stayed up to date throughout the process. Additionally, one researcher worked from the SGL headquarters one day a week to stay in touch and increase approachability.

### 3.2 'How do stakeholders experience their engagement in the process from problem analysis to selecting implementation strategies?'

The themes were the following: strategies to engage, factors influencing engagement and the impact of stakeholder engagement (see Table 4).

#### 3.2.1 Theme 1: Strategies to engage

##### Stakeholders and their roles

Stakeholders experienced a shared responsibility for the success of the implementation and highly valued the inclusion of different perspectives. The care user representative explained she felt her role was to contribute to discussions and speak for care users who otherwise would not be heard. She saw herself as an advocate for other care users.

Care user representative: *'I hear a lot, and I see a lot. Not only "Dit vind ik ervan!"‐related things but in general. I know the people at SGL so I can speak for them.'*

Some stakeholders felt they had dual roles. Care professionals, for example, spoke from both a trainer’s and care professional’s perspective.

Care professional/trainer*: 'What we added was our experiences being a trainer, thus instructing care professionals and using feedback we receive from the care professionals after a training. However, at the same time, being professionals ourselves, we talked about our experiences and struggles. Actually, giving us a double role'*.

The quality of care advisor identified herself as a connector between the project and other projects at SGL. She sometimes thought that the process took too long and that she had to encourage the project group to move forward faster.

Quality of care advisor: *'I think, in a way, mainly practical, being the chain between Zuyd (research) and SGL. So, connecting the things [….] At times, I felt the urge to move forward. You (meaning researchers) took a lot of time, whereas we in an organization, need to think more practical, come on let’s try this!'*

Researchers explained that their role was to share their expertise in implementation and communication. In addition, they also felt responsible for facilitating the group dynamic and balance in power among all stakeholders. To do this, not all researchers attended all meetings, so that there were no situations in which there were more researchers than SGL stakeholders.

##### Use of co‐creation methods and communication supportive strategies

All stakeholders liked the interactive character of meetings. They felt co‐creation methods provided structure and helped them to focus on the meeting's goal. For example, the MoSCoW method used stickers of pre‐defined barriers from the problem analysis, so the participants did not have to formulate their perceived barriers but could use the pre‐defined stickers.

Care professional/trainer: *'When you write it down yourself, then you have to use your own words. Now, I could immediately think about what I felt was important. It is easier because you skip one step, and at the same time, we focus on the meeting's goal'*.

All stakeholders appreciated communication supportive strategies, such as short sentences (max 10 words), use of high frequently used words, one message per sentence, visualizations, pictures and bright colours. They felt meeting reports were useful to prepare meetings and understand the process. However, the care user representative sometimes struggled reading reports due to difficult words or low energy levels.

Care user representative: *'Sometimes it is written down simple, making it easy to read, but other times it is written down difficult, and I have to read it twice. However, this depends on how much energy I have. Whether I am tired or fit…'*.

The fact that the care user did not always understand the meeting reports was not a barrier for her, as she knew that she would prepare the following meeting with the researcher. She appreciated the meetings with the researcher in between the project group meetings. For her, it worked as a reminder and allowed her the opportunity to explain what she meant more deeply.

##### Building relationships

Researchers aimed at building a sustainable relationship with all stakeholders to improve commitment and facilitate in‐depth discussions by offering space for personal stories and not interrupting, especially for the care user representative.

Researcher: '*You really provide her (care user representative) space to speak'*.

Other researchers: '*Yes, and the whole group was conscious that she should have that space'*.

A care professional/trainer explained that she felt the continuous contact with the researchers resulted in a confidential relationship, as she regularly saw the researchers working from SGL headquarters.

Care professional/trainer*: 'It is confidential. You (to the researcher) are now a familiar person because I see you everywhere. This makes it accessible to approach you and talk about certain things'*.

#### 3.2.2 Theme 2: Factors influencing engagement

##### Stimulus of stakeholders to engage

All stakeholders were motivated to engage because they wished others working with the PREM would experience the same benefits of ‘Dit vind ik ervan!’ as they did. Additionally, they not only liked to learn about the quality improvement process but also the opportunity to share experiences of working with ‘Dit vind ik ervan!’.

Care professional/trainer: *'I like being engaged because I am passionate about "Dit vind ik ervan!". And I really like the fact we’re are asked to engage because now I can share my experiences'*.

All stakeholders expressed that they felt that they were an essential contributor to the project group, as they continued to participate throughout the process.

##### Sharing power

All stakeholders reported that they felt free in sharing their experiences and opinions and enjoyed contributing their perspectives. They felt respected and that their opinion was valued.

Care professional/trainer*: 'I might just be "that nurse," so who am I to talk? But with this group, you are not being looked down on, but just respected and if I add something to the discussions I am listened to. That is important!'*

#### 3.2.3 Theme 3: Impact of stakeholder engagement

##### Empowerment of stakeholders

The care user representative experienced that she was empowered to share her experiences and perspectives and enjoyed speaking up for herself.

Care user representative: *'I especially like that I am not only doing this for other care users but also a bit for myself'*.

##### Feeling of a shared responsibility

A care professional/trainer previously experienced being unsupported in dealing with ‘Dit vind ik ervan!’. Due to the project and her engagement, she experienced that implementation success of ‘Dit vind ik ervan!’ was not only her responsibility but a shared responsibility of the entire project group.

Care professional/trainer: *'I have learned that we have a shared responsibility, and you can think about it together, together taking care of improving something. Mainly this feeling of doing it together [….]'*.

##### Learning from one another

Stakeholders explained to have learned from each other and about the complexity of PREM implementation, which needs more than providing the training.

Care professional: *'I really underestimated it. In the beginning, I started teaching the training and felt that was it. Now I know you are not there once you have trained the professionals. Everyone has his own way of working, and it takes a lot of effort to keep everyone on the same page'*.

Researchers learned to adapt their initial aims, which at times were too opportunistic (eg willing to move forward into selecting strategies, whereas stakeholders needed more time to discuss the barriers). Throughout the process, researchers experienced that the process does not just take six meetings, but intense collaboration over the full process period.

Researcher: *'This was a learning process. [….] Because we have had to call them on other occasions as well. You always have to do more stuff. If you would have counted it, this did not just happen in 6 sessions, but during lots of different moments of contact'*.

## 4 DISCUSSION

The aim of this study is to describe a systematic and detailed process of how to get from problem analysis to selecting implementation strategies, to describe how stakeholders can be engaged in that process, and to offer insights into stakeholders’ experiences. We formulated a three‐step process to get from problem analysis to selecting strategies: (a) reaching consensus and prioritizing barriers; (b) categorizing the prioritized barriers and idealization; and (c) composing strategies. Two subthemes continuously play a role in how stakeholders were engaged during the process: communication supportive strategies and continuous contact. The experiences of stakeholder participation resulted in the following themes: ‘stakeholders and their roles’, ‘use of co‐creation methods and communication supportive strategies’, ‘building relationships’, ‘stimulus of stakeholders to engage’, ‘sharing power’, ‘empowerment of stakeholders’, ‘feeling a shared responsibility’ and ‘learning from one another’.

Looking at the identified three‐step process and the way stakeholders were engaged, several principles of design thinking can be recognized (ie user‐centred designs, human‐centred approaches). Design thinking is characterized by empathy for the user, context‐tailored working, and ideation and iteration. It thereby leads to a focus on the needs and preferences of the users. It fits the concept of stakeholder engagement but also complements it by guiding the process of designing products, services and implementation strategies.^33^ To guide the design process, the double diamond model is often used in design thinking.^34^ The two diamonds represent one process of exploring a ‘problem’ more widely and deeply (divergent thinking) and then taking focused action (convergent thinking).^34^ Although not used explicitly in our study, the diamond model has similarities with the three‐step process we applied. Based on the exploration of the implementation barriers in the first study, we focused in this study on prioritizing (step 1) and understanding the root causes (step 2) of these barriers. We thereby defined the ‘problem’ in depth. We then brainstormed and selected implementation strategies to find the answers to the clearly defined problems, in co‐creation with a range of different stakeholders (step 3). The next step will be to iteratively test the strategies on a small scale, which is also a key element of design thinking.^33^

The stakeholders appreciated the use of co‐creation methods throughout the process, for example the MoSCoW method. The MoSCoW method is a prioritizing tool, which we used to prioritize all identified barriers in the problem analyses. This was experienced as useful by stakeholders, because pre‐defined barriers limited space for interpretation, which made the decisions easier. Stakeholders felt as if they had more time to rank the statements and that the method helped them focus on the meetings’ tasks. Other studies found a similar dynamic, using co‐creation methods in the engagement of care users, as co‐creation methods could provide micro‐level insights into the patient (care user)‐centred services.^23^, ^35^

In our study, a great focus was also on using communication supportive strategies throughout the whole process to facilitate stakeholder engagement. Previous studies acknowledge the significant role of using communication supportive strategies in order to support communication‐vulnerable care users, such as the use of short sentences (max 10 words per sentence), use of frequently used words and one message per sentence.^15^, ^36^, ^37^ From our study, we learned that not only the care user representative but also other stakeholders felt supported through the use of a variety of communication supportive strategies during the process. The communication supportive strategies helped stakeholders to prepare meetings and to understand the complexity of the implementation. A possible explanation for this finding is that stakeholders had no similar previous experience with the process of implementation and so felt assisted by a communication‐friendly way of working.

Moreover, the importance of building a relationship among stakeholders seemed fundamental. The continuous contact in and between regular meetings made stakeholders feel listened to and researchers approachable, which helped build trust and sustainability in stakeholder engagement. This outcome is confirmed by studies in which an approachable relationship and frequent contact benefit the engagement of stakeholders.^38^ Relations were established by regular interaction with the research team and site visits to locations for meetings with employees. Additionally, a close relationship helps with introductions into and learning about the context in which research takes place.^23^, ^38^, ^39^

In addition, the inclusion of stakeholders from multiple levels of the organization facilitated gathering insights from the different stakeholders’ perspectives and was appreciated by all stakeholders. However, having an inclusive stakeholder group demands extra time (eg preparing communication‐friendly reports, meeting with the care user representative and working from SGL location). Dobbins et al^38^ also found that inclusive stakeholder engagement is complex and time‐consuming because of the ongoing support, customized approaches and having regular contact with stakeholders. Nevertheless, including all types of stakeholders leads to a broad knowledge input,^8^ which potentially improves the sustainability of the intervention, as shown in previous studies.^10^, ^11^ Moreover, we showed that valuable inclusion of care users and care professionals is possible if factors, such as preparation, communication‐friendly presentations and reports, and relationship building, are taken into account.

### 4.1 Strengths and limitations

A strength is that we used both inductive and deductive analyses for the two research questions. We used the inductive approach to explore the process of getting from problem analysis to selecting implementation strategies, as limited knowledge was available on this topic. Furthermore, we used a deductive approach for exploring experiences with stakeholder engagement, as stakeholder engagement is a well‐defined concept. In addition, the analysis framework we used was based on a comprehensive scoping review about stakeholder engagement by Camden et al^5^ The framework supported us in developing the interview guide and guided the structured analysis. Another strength is that we included all participating researchers as stakeholders in the data collection and so explored their experiences with the process, as well. Although researchers often participate in stakeholder groups, to our knowledge, little reflection occurs upon researchers’ experiences with their engagement, which potentially hinders the translation of lessons learned.

A limitation of this study is the relatively small representation of care user representatives during the process. We initially included three care user representatives. However, care user representatives in this sector are fragile and we did not foresee that two out of three care users representatives would drop out. We did not opt for replacing the two, as initially their medical conditions seemed to be temporary. We could not find a publication that specified a number of stakeholders needed for stakeholder engagement in research.^18^, ^19^, ^25^ Nevertheless, in the future, we would aim for including more care users at the beginning or opting for the inclusion of third parties (eg informal (spousal) caregivers, friends or family of care users), who would then use their experience to reflect from a care user perspective.^25^ They bring in their experience to reflect from a care user perspective. This will strengthen the stakeholder engagement process and reinforce co‐creation.^23^

### 4.2 Implications for practice and research

Our study has implications for future research and can guide those who aim to implement interventions in health care. We think that:

- The presented three‐step process and the practical description of the application of co‐creation methods within this process can support implementers in applying the process of getting from problem analysis to selecting strategies while engaging stakeholders. Further research in different health‐care settings may help researchers and other stakeholders discover the transferability and potential further use of the three‐step process.
- Implementation science can learn from tools used in design thinking, such as the co‐creation methods, as they help visualize and design implementation strategies while engaging stakeholders.
- Communication supportive tools can be used to support not only communication‐vulnerable stakeholders but also other relevant stakeholders.
- Continuous contact with involved stakeholders is of high importance as this supports relationship building. This can be accomplished by preparing meetings together, reporting about meetings, but also by being visible in the organization.
- Composing an inclusive stakeholder group might be more time‐consuming and sensitive to the dropout of vulnerable care users. Nevertheless, our study shows the possibilities for inclusive stakeholder engagement when taking into account the inclusion of more care users or third parties and using co‐creation methods tailored to stakeholders’ capacities.

## 5 CONCLUSION

The presented three‐step process and the described co‐creation methods might offer future implementers guidance for organizing the process from problem analysis outcomes to selecting implementation strategies by using valuable stakeholder engagement. Valuable stakeholder engagement can be facilitated by using supportive communication strategies (not only for people who are communication vulnerable), being in continuous contact with all stakeholders, and taking time to include all relevant stakeholders, as well as building sustainable relationships. Applying these strategies, the stakeholders in our study experienced a shared responsibility for the implementation success and described their experience of engagement as relevant and valuable.

## CONFLICT OF INTEREST

None declared.

## Data Availability

The datasets generated during and/or analysed during the current study are available from the corresponding author upon reasonable request. The data that support the findings of this study are available on request from the corresponding author. The data are not publicly available due to privacy or ethical restrictions.
